# Knowledge; an Introductory Address, Delivered at the Bristol Athenæum, October 5, 1846

**Published:** 1847-01

**Authors:** 


ART. XVIII. — *Knowledge; an Introductory Address, delivered at the Bristol Athenæum, October* 5, 1846. By JOHN ADDINGTON SYMONDS, M.D.—*London,* 1846. 12mo, pp. 48.

"WHAT is knowledge? How is it obtained? What are its principal divisions? In what spirit should it be pursued?" are the questions which Dr. Symonds has essayed to answer in this address; and notwithstanding its brevity they are most clearly and attractively handled. This lecture, in common with all that proceeds from Dr. Symonds's pen, evinces the high intellectual and moral cultivation, the elegant taste, and the comprehensive acquirements of its accomplished author; and we strongly recommend its perusal to our younger friends, as likely to give them a definite apprehension of the objects of their pursuit, to stimulate their zeal in the laborious studies they have undertaken, and to elevate their ideas of the real value and ultimate purposes of that which they seek to acquire.

